# Preventive effects of *phenylethanol glycosides* from *Cistanche tubulosa* on bovine serum albumin-induced hepatic fibrosis in rats

**DOI:** 10.1186/s40199-015-0135-4

**Published:** 2015-12-09

**Authors:** Shu-Ping You, Jun Zhao, Long Ma, Mukaram Tudimat, Shi-Lei Zhang, Tao Liu

**Affiliations:** Department of Toxicology, School of Public Health, Xinjiang Medical University, No. 393 Xinyi Road, Urumqi, 830011 Xinjiang Uyghur Autonomous Region China; Key Laboratory for Uighur Medicine, Institute of Materia Medica of Xinjiang, Urumqi, 830004 China; No. 140 Xinhua South Road, Tianshan District, Urumqi, 830000 Xinjiang Uyghur Autonomous Region China

**Keywords:** Hepatic fibrosis, Cistanche tubulosas, Phenylethanol glycoside, Chemokine BSA, Prevention and therapy

## Abstract

**Background:**

*Cistanche tubulosa* is a traditional Chinese herbal medicine that is widely used for regulating immunity. Phenyl ethanol glycosides (CPhGs) from this plant are the primarily efficacious materials. This aim of this study was to evaluate the preventive and therapeutic effects of CPhGs on BSA-induced hepatic fibrosis in rats and related molecular mechanisms involving hepatic stellate cells. Biejiarangan (BJRG), another traditional Chinese herbal medicine, was used as a positive control.

**Methods:**

In in vivo experiments, 75 SD rats were randomly divided into 6 groups: normal (distilled water-treated), model (BSA-treated), positive drug (BSA-treated + BJRG 600 mg/kg/day), and BSA-treated + CPhGs (125, 250, and 500 mg/kg/day) groups. The liver and spleen indices, serum levels of aspartate aminotransferase (AST), alanine aminotransferase (ALT), hexadecenoic acid (HA), laminin (LN), type III procollagen (PCIII), type IV collagen (IV-C), hydroxyproline (Hyp), and transforming growth factor *β*_1_ (TGF-*β*_1_) were measured in rat livers. Histopathological grades for liver fibrosis were assessed for each group using H&E and Masson’s trichrome staining. The expression of TGF-*β*_1_, collagen I (Col-I) and collagen III (Col-III) were determined by an immunohistochemical staining method. These effects were further evaluated in vitro by determining expression levels of NF-κB p65 and Col-I by quantitative real-time PCR analyses. Col-I protein expression was also examined by western blotting.

**Results:**

All dose groups (125, 250, and 500 mg/kg/day) of CPhGs significantly reduced the liver and spleen index, decreased ALT, AST, HA, LN, PCIII, IV-C serum levels, TGF-*β*_1_ content (*P* < 0.01, *P* < 0.01, and *P* < 0.01), and Hyp content. CPhGs also markedly alleviated the swelling of liver cells and effectively prevented hepatocyte necrosis and inflammatory cell infiltration. Immunohistochemical results showed that CPhGs significantly reduced the expression of TGF-*β*_1_ (*P* < 0.01, *P* < 0.01, and *P* < 0.01)_,_ Col- I, and Col-III. The in vitro effects of CPhGs (100, 75, 50, and 25 ug/ml) on HSC-T6 showed that CPhGs significantly reduced mRNA expression of NF-κB p65 and Col-I, and CPhGs also downregulated Col-I protein expression.

**Conclusions:**

CPhGs have a significant anti-hepatic fibrosis effect, and may be used as hepatoprotective agents for treatment of hepatic fibrosis.

## Background

Hepatic fibrosis is a wound healing response to severe liver injury that occurs in the pathogenesis of chronic hepatitis induced by various factors. These factors are viral infection, alcohol abuse, cholestasis, and metabolic and autoimmune diseases [1–3]. Progressive accumulation of extracellular matrix (ECM) and decreased remodeling disrupt the normal architecture of the liver, resulting in hepatic fibrosis [4]. Hepatic fibrosis is critical in chronic liver disease, and often develops into irreversible cirrhosis and carcinogenesis. At present, there are no methods or effective drugs for the treatment of hepatic fibrosis. Therefore, it is urgent to find an anti-hepatic fibrosis drug that will attenuate the progression of liver injury to fibrosis and cancer.

*Cistanche tubulosa* W (of the family Orobanchaceae) is a parasitic plant that is widely grown in the southern region of Xinjiang in China [5]. People usually use it to invigorate the kidneys, nourish the blood, relax the bowel, and delay senescence. It is officially listed in the Chinese Pharmacopoeia [6]. *C. tubulosa* contains a variety of active components. These include phenyl ethanol glycosides (CPhGs), iridoids, and polysaccharides. As one of many active components in *C. tubulosa*, CPhGs have exhibited convincing antioxidant, anti-fatigue, neuroprotective, and anti-inflammory effects in both in vivo and in vitro studies [7]. In recent years, it has been reported that CPhGs have hepatoprotective effects. Potential mechanisms underlying these effects are scavenging of free radicals, protection of hepatic membranes, immunoregulation, inhibition of apoptosis, inhibition of the expression of HBsAg and HBeAg, and inhibition of HBV DNA replication and others [8–11]. However, few studies in the literature address the anti-hepatic fibrosis effect of GPhCs. Therefore, this study aimed to investigate the anti-hepatic fibrosis effect of GPhCs by using a model of bovine serum albumin (BSA) induced hepatic fibrosis in rats. Related molecular mechanisms were investigated in HSC-T6 cells.

## Methods

### Chemicals and reagents

A Hydroxyproline kit (Alkaline hydrolysis) (Lot: 20140616) was purchased from Nanjing Jiancheng Bioengineering Institute (China). Rat TGF-*β*_1_ sandwich ELISA kits (Lot: 238240615) was purchased from Lianke Biotech Co., Ltd. (China). Rabbit Anti-Collagen I antibody (Lot: 140619), Rabbit Anti-Collagen III antibody (Lot: 980788 W), and Rabbit Anti-TGF-β_1_ antibody (Lot: 140619) were provided by Beijing Biosynthesis Biotechnology Co., LTD (China). Enclosed with normal sheep serum (working fluid) (Lot: WP141214), PV-6000 (Lot: WK141225), and DAB kit (Lot: K136621D) were purchased from Zhongshan Golden Bridge Bio-tech Co., Ltd. (China). Detection of primary antibodies was performed by using 2. Antibody solution (Alk-Phos. Conjugated, Anti-rabbit) and 2. Antibody solution (Alk-Phos. Conjugated, Anti-mouse) (lot: 272387) purchased from Invitrogen company (USA).

Bovine serum albumin (BSA) (Lot: SLBG8239V) was purchased from Sigma (USA). Before use, BSA was prepared at 18 g/L in normal saline, the bacteria removed by filtration, and BSA stored at 4 °C. Freund’s incomplete adjuvant containing 1 g of lipid from sheep hair (Lot: AF0220LA14, Shanghai yuanye Bio-Technology Co., Ltd. (China,) was mixed with 2 g liquid paraffin (Lot: 20130815, Tianjin Fuyu Fine Chemical Co., Ltd. (China,), sterilized in a steam autoclave, and stored at 4 °C . The positive drug BJRG was obtained as Biejiarangan tablets from Inner Mongolia Furui Medical Science Co., Ltd. (China). BJRG drug stocks were prepared by dissolving BJRG tablets in distilled water at a concentration of 600 mg/kg.

### Plant materials

*C. tubulosa* (Orobanchaceae family) was purchased from the Minfeng region of Xinjiang of China. The material was authenticated by researcher Jun Zhao, Key Laboratory for Uighur Medicine, Institute of Materia Medica of Xinjiang. Voucher specimens were deposited in the Institute of Materia Medica of Xinjiang.

### Preparation of CPhGs

Dried and sliced rhizomes of *C. tubulosa* (6.0 kg) were consecutively extracted under reflux three times with 70 % ethanol, and the solvent was removed to yield the ethanol extract. Ethanol extracts were purified by using AB-8 resin to obtain the phenyl ethanol glycosides (CPhGs). Stock solutions of CPhGs used for different dose groups in animal studies (500 mg/kg, 250 mg/kg, and 125 mg/kg, respectively) were dissolved in 0.5 % (5 g/L) carboxymethyl cellulose (sodium salt).

### Quantification of CPhGs

The contents of two components (echinacoside and acteoside) in the CPhGs were determined by HPLC using a previously reported method (Zhang et al. 2004) [12]. HPLC was performed by using a Shimadzu LC-10A HPLC equipped with a UV detector. The HPLC column was a Phenomenex Gemini ODS column (250 × 4.6 mm, 5 μm). The isocratic mobile phase consisted of methanol-acetonitrile-1 % acetic acid (15:10:75, v/v/v). Elution was for 40 min and the flow rate was kept at 0.6 mL/min. Column temperature was kept constant at 30 °C. UV detection was at 334 nm.

### Animals and HSC-T6 cell line

[Grade SPF] healthy adult male Sprague–Dawley (SD) rats (180–220 g) were purchased from Xinjiang Medical University Animal Center, License No.: SCXK (New) 2011–0004. Rats were fed specific-pathogen free (SPF) chow. All of the procedures related to the animal experiments were approved by the Animal Ethics Committee of First Affiliated Hospital of Xinjiang Medical University. Rats were housed in cages under controlled environmental conditions (25 °C and a 12 h light/dark cycle) and had free access to standard rat pellet food and tap water. Rats were acclimated before treatment.

An immortalized rat hepatic stellate cell line, HSC-T6, was obtained from Wuhan Procell Gene Bio-technology Co., LTD. (Wuhan, China). HSC-T6 cells were cultured in Dulbecco’s Modified Eagle’s Medium (High Glucose) (DMEM, Beijing, China) supplemented with 10 % fetal bovine serum (Gibco, South America), 100 IU/ml penicillin and 100 *μ*g/ml streptomycin (Beijing, China) in a humidified incubator at 37 °C with 5 % CO_2_.

### BSA-induced liver injury and treatments

Seventy-five SD rats were randomly divided into six groups: Normal (distilled water-treated), model (BSA-treated), positive drug (BSA-treated + BJRG 600 mg/kg/day), and BSA-treated + CPhGs (125, 250, 500 mg/kg/day) groups. BSA-treated + CPhGs (125, 250, 500 mg/kg/day) groups had 13 rats in each group, and other group had 12 rats in each group. The BSA-induced liver injury model is divided into primary sensitization followed by immunological attack) [13]. Except for the normal group, the other groups were administered multiple subcutaneous injections with 0.5 ml (9 mg/ml) BSA Freund’ incomplete adjuvant on day _1_, day _15_, day _22_, day _29,_ and day _36_ for primary sensitization_._ Seven days after the fifth injection, blood was obtained through the rat retinal vein plexus and tested for serum albumin antibodies. BSA antibody in rat serum was detected by a double agar diffusion method. The attack injection was performed by administering 0.4 ml of BSA in normal saline was through the caudal vein in BSA antibody-positive rats twice a week for ten times. The concentrations and times of injections were 5.00, 5.50, 6.00, 6.50, 7.00, 7.50, 8.50, 9.00, 9.50 and 10.00 g/L at day _46_, day _50_, day _53_, day _57_, day _60_, day _64,_ day _67_, day_71_, day _74_ and day _78_, respectively. In the normal group, normal saline was used for immunological primary (sensitization) and secondary (attack) injections instead of BSA, and other conditions were the same as those in the model group.

The normal group was orally administered distilled water with dose 10 ml/kg/day. The model group was orally administered 10 ml/kg/day 0.5 % CMC-Na solution. The positive drug group was orally administered 600 mg/kg/day BJRG. The BSA-treated + CPhGs (125, 250, and 500 mg/kg/day) groups were orally administered 125, 250, and 500 mg/kg/day CPhGs, respectively. Daily dosing rats continued for two weeks after the last injection.

After the experimental period, rats were fasted for 12 h prior to 10 % chloral hydrate and then immediately euthanized.. Serum samples were collected from each rat and immediately used. Livers were harvested for two purposes: (1) preservation in liquid nitrogen for Hyp kits and (2) fixation in 10 % formaldehyde for histological and immunohistochemical examinations. The entire duration of the animal studies was 93 days.

### Liver and spleen indices

The liver and spleen were dissected by laparotomy and washed with 4 °C normal saline. After absorbing excess water with filter paper, the liver and spleen were weighed to calculate the corresponding indices: Relative organ weight = organ mass (g)/individual body mass (g) × 100 % [14].

### Analysis of markers of liver fibrosis

The protective effect of CPhGs against BSA-induced liver injury was evaluated by measuring ALT and AST (Mindray automatic biochemical analyzer,A086A0182). The development of liver fibrosis and effects of treatment were determined by examining HA, LN, PC III, and IV-C (Chemiluminescence analyzer,TaiGeKeXin, MP2808).

### Hyp content and TGF-*β*_1_ analysis

The level of Hyp in liver tissue was determined by a spectrophotometric method according to the kit’s instructions. The level of Hyp was expressed as Hyp (μg)/protein (mg). Hyp (μg/mg) = (Measured _OD_- blank _OD_)/(standard _OD_- blank _OD_) × 5 (μg/ml) × 10/tissue wet weight (ml/mg). The hepatic concentration of TGF-*β*_1_ was detected by using ELISA kits according to the manufacturer’s instructions. The inhibitory effect of CPhGs on fibrosis was confirmed by the expression levels of TGF-*β*_1_.

### Histopathological examination

The liver tissue in the same part of the left lobe was resected and fixed in 10 % formaldehyde solution. Tissues were stained with conventional H&E and Masson’s trichrome staining to observe histopathological changes under a light microscope. Expression of collagen type I, collagen type III, and TGF-β_1_ in liver tissues was analyzed by immunohistochemical staining [15].

Semi-quantitative immunohistochemistry was performed according to reported methods [16, 17]. The following classifications were used for TGF-β_1_ positive cells: “-” indicates almost no expression, 2^0^ = l; “+” indicates positive cells individually gathered in the lesion area, 2^1^ = 2; “++” indicates positive cells in small groups gathered around the lesion area, 2^2^ = 4; “+++” indicates dispersed positive cells expressed, as 2^3^ = 8. The results represent a hierarchical integration. The collagen type I and collagen type III chromogenic degree and scope were converted into CRI for statistical analysis (chromogenic degree × chromogenic range). The chromogenic degree is divided into weak “+”, moderate “++” and strong “+++”. The chromogenic range is divided into: “+”, its chromogenic range < view 1/4; “++”, its chromogenic range of vision/4-2/4; “+++”, its chromogenic range of vision 2/4-3/4; “++++”, its chromogenic range > 3/4. Blinded scoring was performed by two people where “+” is 1; “++” is 2, “+++” is 3, and “++++” is 4. Three microscope observational fields (magnification × 200) were randomly selected for each section, with the average level of the samples used as a semi quantitative level.

### Cell experiments

HSC-T6 cells were plated in a 96-well plate. Initially, cells were cultured with DMEM containing 10 % FBS for 48 h. The medium was then replaced with DMEM without FBS to starve the cells for 12 h. The cells were then cultured with DMEM that contained 5.0 ng/mL TGF-β_1_ (without FBS) for 24 h. Finally, different concentrations of CPhGs (100ug/ml, 50ug/ml, and 25ug/ml), acteoside (6 ug/ml, 3 ug/ml, and 1.5 ug/ml), and echinacoside (500ug/ml, 250ug/ml, and 125ug/ml) were carried out in the plate in qudruplicate wells and incubated for 48 h.

### Real-time PCR analysis

The mRNA expression level of NF-κB, p65, and collagen I were determined by real-time PCR. To determine mRNA expressions in HSC-T6 cells, the cells (4 × 10^5^ cells) were seeded in six-well plates with 3 mL DMEM with 10 % FBS and incubated overnight at 37 °C and 5 % CO_2_, after which the cell culture media were changed to serum-free DMEM. Next, CPhGs (100 ug/ml, 50 ug/ml, and 25 ug/ml), acteoside (6 ug/ml, 3 ug/ml, and 1.5 ug/ml), and echinacoside (500 ug/ml, 250 ug/ml, and 125 ug/ml) were added to the wells. After 48 h of incubation with CPhGs or monomeric compositions, total RNA was extracted using TRIzol reagent (Invitrogen, USA) and agitated vigorously with chloroform for 15 s. After sitting at room temperature for 3 min, the lysate was centrifuged at 12,000 × g for 15 min at 4 °C. RNA in the aqueous phase was precipitated with isopropanol, and the upper aqueous phase was transferred to a new microcentrifuge tube. RNA was precipitated by adding 0.75 % ethanol, after which the microcentrifuge tube and centrifuged at 12,000 × g at 4 °C for no more than 5 min. The supernatant was removed and the RNA was dried at room temperature for 5–10 min. Specific sets of primers (Sangon, Shanghai, China) that were used for amplification of rat β-actin [GenBank: NM_031144.3], Collagen I [GenBank: NM_ 053304.1], and NF-κB p65 [GenBank: NM_199267.2] genes were designed using Batch Primer 3. The forward (fw) and reverse (rv) primers were as follows: Collagen I (fw: GGA GAG AGC ATG ACC GAT GG, rv: GGG ACT TCT TGA GGT TGC CA), NF-κB p65 (fw: CAT ACG CTG ACC CTA GCC TG, rv: TTT CTT CAA TCC GGT GGC GA), β-actin (fw: TAA GGC CAA CCG TGA AAA GAT G, rv: AGA GGC ATA CAG GGA CAA CAC A). Results were normalized to the mRNA of the housekeeping gene β-actin as an internal control and are presented as relative mRNA levels.

Reactions were performed with 8 μL iQ SYBR Green Supermix, 1 μL 10 pM primer pair, 8.5 μL distilled water, and 2.5 μL cDNA. Each polymerase chain reaction was performed under the following conditions: 95 °C for 3 min, then 40 cycles of 10 s at 95 °C, 30 s at 55 °C, and 10 s at 55 °C – 95 °C for extension, followed by a single fluorescence measurement. The final results were described with the relative values (2^-ΔΔCt^). Calculation and analysis were performed by the iQ5 Real Time PCR Detection System.

### Western blot analysis

Collagen I (Abcam, Cambridge, UK, Art No: ab34710) protein expression levels were determined by Western blotting [with β-actin (Lot: 60008-1-lg; Proteintech, China) as a housekeeping control. Whole cell extracts were prepared using Radioimmunoprecipitation assay (RIPA) (Thermo Scientific, USA) buffer with 1 % Halt protease inhibitor cocktail (Thermo Scientific, USA) and 1 % Halt phosphatase inhibitor cocktails (Thermo Scientific, USA). The protein concentration was measured and quantified by the Bradford method [18]. Protein (10–50 ug) was separated on a 10 % SDS-PAGE gel and transferred to PVDF membranes (Millipore, USA). Membranes were blocked for 1 h at room temperature with 5 % BSA, and the primary antibodies (Anti-Collagen I antibody, 1:200 dilution or mouse mAb of β-actin, 1:5000 dilution) were incubated at 4 °C overnight. The corresponding Alk-Phos. conjugated secondary antibodies were incubated at room temperature. Finally, the membranes were washed three times with 1 × Tris–HCl saline with 0.1 % Tween 20, and signals were scanned and visualized by GEL DOC XR Imaging System (Bio-Rad). Densitometric analysis was performed on the proteins of interest and normalized to β-actin by GEL DOC Image Studio software (Bio-Rad). β-actin was used as the internal control.

### Statistical analysis

The Shapiro-Wilk normality test and Levene’s variance homogeneity test were applied to verify normality and homogeneity of variance. Analysis of variance (ANOVA) followed by Tukey’s post hoc test was used to identify statistical differences in homogeneous, normally distributed data. The Kruskal-Wallis non-parametric test was used to analyze data not normally distributed or homogeneous. Results were expressed as mean ± SD. Significance was set at *P* < 0.05. All data were analyzed by SPSS 16.0 software (Xinjiang Medical University).

## Results

### Quantitative determination of CPhGs

**Fig. 1:**
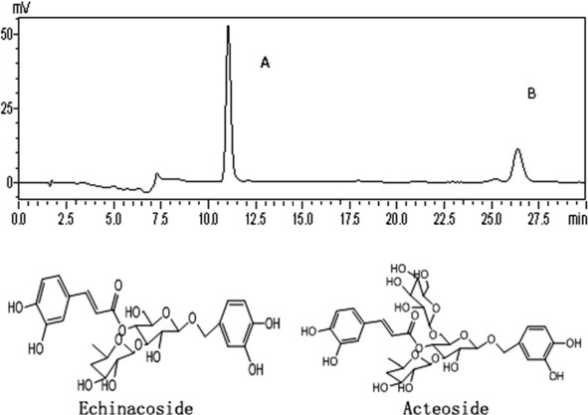
The HPLC analysis of CPhGs and two phenylethyl alcohol glycosides, echinacoside and acteoside

CPhGs in *C. tubulosa* contains two phenylethyl alcohol glycosides, echinacoside and acteoside, and their contents in the CPhGs were determined by HPLC analysis (Fig. 1) to be 42.71 ± 0.42 % and 14.27 ± 0.18 %, respectively.

### Liver and spleen indices

As Table 1 was shown, the liver and spleen indices of the model group were elevated significantly [*P* < 0.01, *P* < 0.05]. The liver and spleen indices of the positive drug BJRG and CPhGs at different dose groups were significantly reduced compared with the model group [*P*_Liver_ = 0.004, *P*_Liver_ = 0.003, *P*_Liver_ = 0.004, *P*_Liver_ = 0.005; *P*_Spleen_ = 0.017, *P*_Spleen_ = 0.027, *P*_Spleen_ = 0.024, *P*_Spleen_ = 0.070, respectively]. The liver and spleen indices were lower than those of the model group.

**Table 1:** Effects of CPhGs on the liver and spleen indices of hepatic fibrosis rats

Group	Dose (mg/kg/day)	n	Liver index (%)	Spleen index (%)
Normal	—	12	2.21 ± 0.077^**^	0.15 ± 0.010^**^
Model	—	11	2.65 ± 0.245	0.19 ± 0.025
BJRG	600	10	2.21 ± 0.253^**^	0.17 ± 0.013^*^
CPhGs	500	12	2.32 ± 0.153^**^	0.16 ± 0.025^*^
	250	9	2.40 ± 0.150^**^	0.16 ± 0.017^*^
	125	10	2.39 ± 0.103^**^	0.16 ± 0.033

Values are expressed as mean ± SD, *n* = Survival number of animals

^*^ *P* < 0.05, Compared with the model group;^**^ *P* < 0.01, Compared with the model group

### Effects of CPhGs on ALT, AST activities and liver fibrosis markers

In the present study, the serum levels of the hepatic enzymes AST and ALT were significantly increased in the model group, reflecting hepatocellular damage in BSA-induced liver fibrosis rats. However, the experiments showed that treatment with BJRG (600 mg/kg) and CPhGs (125, 250, and 500 mg/kg) significantly reduced AST [*P*_AST_ < 0.001, *P*_AST_ < 0.001, *P*_AST_ < 0.001, *P*_AST_ < 0.001, respectively] and ALT [*P*_ALT_ = 0.117, *P*_ALT_ = 0.139, *P*_ALT_ = 0.189, *P*_ALT_ = 0.255, respectively] levels in hepatic fibrosis rats. (Table 2).

**Table 2:** Effects of CPhGs on the serum AST, ALT activities of hepatic fibrosis rats

Group	Dose (mg/kg/day)	n	ALT (U/L)	AST (U/L)
Normal	—	12	38.12 ± 2.789^**^	77.82 ± 16.675^**^
Model	—	11	46.26 ± 6.904	173.27 ± 27.389
BJRG	600	10	39.68 ± 6.716	104.65 ± 8.891^**^
CPhGs	500	12	39.69 ± 7.048	105.23 ± 8.865^**^
	250	9	39.98 ± 5.476	106.60 ± 20.270^**^
	125	10	40.95 ± 3.920	119.72 ± 25.368^**^

Values are expressed as mean ± SD, *n* = Survival number of animals

^**^ *P* < 0.01, Compared with the model group

The levels of HA, LN, PC and IV-C in model rats were significantly increased [*P*_HA_ < 0.001, *P*_LN_ < 0.001, *P*_PCIII_ = 0.002, *P*_IV-C_ < 0.001, respectively]. Compared with the model group, the levels of HA [*P*_HA_ = 0.009, *P*_HA_ = 0.007, *P*_HA_ = 0.009, *P*_HA_ = 0.023, respectively], LN [*P*_LN_ = 0.011, *P*_LN_ = 0.004, *P*_LN_ = 0.026, *P*_LN_ = 0.069, respectively], PC III [*P*_PCIII_ = 0.006, *P*_PCIII_ = 0.067, *P*_PCIII_ = 0.136, *P*_PCIII_ = 0.296, respectively], and IV-C [*P*_IV-C_ < 0.001, *P*_IV-C_ < 0.001, *P*_IV-C_ < 0.001, *P*_IV-C_ < 0.001, respectively] in rats were markedly decreased by BJRG (600 mg/kg) and CPhGs at different dose groups (125, 250, and 500 mg/kg) (Table 3).

**Table 3:** Effects of CPhGs on the serum HA, LN, PCIII, IV-C activities of hepatic fibrosis rats

Group	Dose (mg/kg/day)	n	HA (mg/l)	LN (mg/l)	PCIII (mg/l)	IV-C (mg/l)
Normal	—	12	98.67 ± 15.798^**^	21.27 ± 3.003^**^	12.69 ± 6.525^**^	1.99 ± 0.691^**^
Model	—	11	116.08 ± 7.683	29.38 ± 4.716	33.87 ± 16.336	8.10 ± 0.369
BJRG	600	10	106.85 ± 7.650^**^	23.35 ± 3.106^*^	21.09 ± 7.113	2.16 ± 0.603^**^
CPhGs	500	12	104.97 ± 9.139^**^	23.91 ± 5.945^*^	23.58 ± 8.815	2.52 ± 0.464^**^
	250	9	106.19 ± 6.250^**^	24.12 ± 3.593^*^	24.07 ± 11.859	3.10 ± 0.692^**^
	125	10	108.11 ± 8.261^*^	25.41 ± 4.925	21.79 ± 7.877	3.39 ± 0.980^**^

Values are expressed as mean ± SD, *n* = Survival number of animals

^*^ *P* < 0.05, Compared with the model group; ^**^ *P* < 0.01, Compared with the model group

### Hyp content and TGF-*β*_1_

Collagen content was also detected by measuring Hyp levels in liver tissue. As shown in Table 4, the mean Hyp level in the model group was significantly higher than the normal group, but it was markedly decreased in the BJRG group and the different CPhGs dose groups.

**Table 4:** Effects of CPhGs on the Hyp content and TGF-*β*_1_ of hepatic fibrosis rats

Group	Dose (mg/kg/day)	n	Hyp (μg/mg)	TGF-*β*_1_ (ng/ml)
Normal	—	12	125.61 ± 57.118^*^	16.58 ± 2.814^**^
Model	—	11	196.70 ± 82.545	31.24 ± 6.726
BJRG	600	10	129.57 ± 36.806	20.16 ± 4.638^**^
CPhGs	500	12	132.77 ± 50.705	21.75 ± 3.711^**^
	250	9	134.99 ± 64.824	22.93 ± 3.576^**^
	125	10	139.14 ± 41.352	23.03 ± 4.212^**^

Values are expressed as mean ± SD, *n* = Survival number of animals

^*^ *P* < 0.05, Compared with the model group; ^**^ *P* < 0.01, Compared with the model group

The hepatic concentration of TGF-*β*_1_ of the model group in rats was significantly increased [*P* < 0.001]. Compared with the model group, TGF-*β*_1_ levels of the positive drug and the different CPhGs dose groups were markedly decreased [*P*_TGF-*β*1_ < 0.001, *P*_TGF-*β*1_ < 0.001, *P*_TGF-*β*1_ < 0.001, *P*_TGF-*β*1_ < 0.001, respectively]. The results are summarized in Table 4.

### Histopathological examination

Observations of normal liver tissue sections stained with H&E and Masson’s trichrome exhibit distinct hepatic lobules and hepatic sinusoids. The liver tissue structure in the model rats was disordered, and the liver tissue and hepatic sinusoids were replaced by a large amount of connective tissue. However, more normal cytoarchitechture and less connective tissue were detected in the treatment group than those in the model group.

### H&E staining

**Fig. 2:**
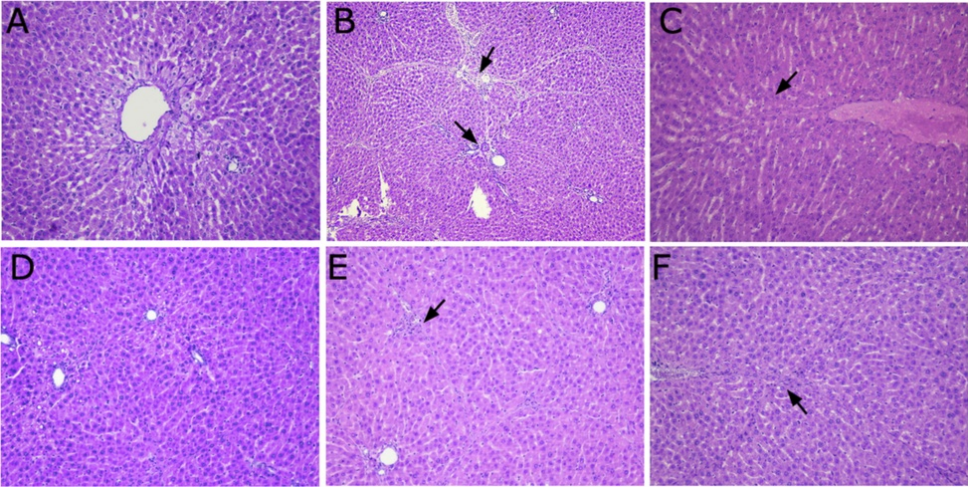
H&E staining in BSA-induced hepatic fibrosis rats (H&E stain, magnification × 200). **a** normal group; **b** model group; **c** BJRJ, 600 mg/kg; **d** CPhGs, 500 mg/kg; **e** CPhGs, 250 mg/kg; **f** CPhGs, 125 mg/kg

In the normal group, hepatic lobule structural integrity without abnormal portal areas and hepatic sinusoids was observed. The hepatic cords were arranged in an orderly fashion, with the core round and clear. The nuclei are located in the central of the cell, with abundant cytoplasm. Only the portal area has a small amount of fibrous tissue (Fig. 2a).

In the model group, the lobular structure was severely damaged. Liver cells showed mild watery degeneration, mostly ballooning degeneration and/or fatty degeneration. The formation of inflammatory cell infiltration, extensive fibrous tissue hyperplasia, the formation of a large number of fibrous septum, split lobules, and significant liver cells proliferation were also observed. These observations confirmed the success of the establishment of the rat immune injury animal model of hepatic fibrosis (Fig. 2b).

Compared with the model group, there was reduction in inflammatory cell infiltration in the different CPhGs. Also observed were less necrosis and fatty degeneration of liver cells as well as alleviation of fibrosis. CPhGs significantly mitigated the pathology of BSA-induced hepatic fibrosis in rats, alleviated the swelling of liver cells, and effectively prevented hepatocyte necrosis and inflammatory cells infiltration, suggesting that CPhGs exert protective effect on BSA -induced rat hepatic fibrosis. (Fig. 2c–f).

### Masson’s trichrome staining

**Fig. 3:**
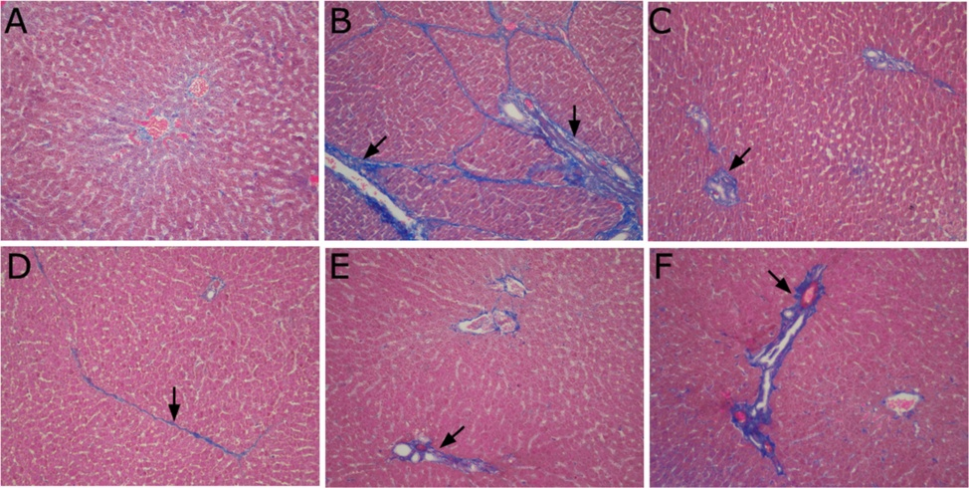
Masson’s trichrome staining in BSA-induced hepatic fibrosis in rats (Masson’s trichrome stain, magnification × 200). **a** normal group; **b** model group; **c** BJRJ, 600 mg/kg; **d** CPhGs, 500 mg/kg; **e** CPhGs, 250 mg/kg; **f** CPhGs, 125 mg/kg

In the normal group, the liver tissue was normal. The hepatic portal area showed a small amount of blue collagen fibers, and the liver tissue was normally structured (Fig. 3a). Compared with the liver tissue of the normal group, fibrous tissue proliferated by the central leaflet and expanded into the liver parenchyma. Collagen fibers extended and linked and enveloped the entire lobule and surrounded the central vein. These effects, along with hepatocyte fibrosis, lobular structural damage, periportal fibrosis, and pseudolobule formation (Fig. 3b) provided evidence that the model was established.

Compared with the model group, the collagen fibers in the positive drug control group were mildly extended outward from the peripheral portal area (Fig. 3c). Compared with the model group, the collagen fibers in the different CPhGs dose groups were significantly reduced, fiber proliferation was inhibited, and proliferation of fibrous tissue within the liver parenchyma significantly reduced. These results suggested that CPhGs protected rats from BSA-induced hepatic fibrosis (Fig. 3d–f).

### Immunohistochemical staining

Importantly, the expression of collagen type I and collagen type III play essential roles in the development of hepatic fibrosis, and their generation and deposition in the liver tissue could serve as an important determinant of the anti-hepatic fibrosis efficacy. The results are summarized in Table 5.

**Table 5:** The immunohistochemical staining intensity of *Collagen type I, Collagen type III and TGF-β1* i n BSA-induced hepatic fibrosis rats

Group	Dose (mg/kg/day)	*n*	Collagen type I				Collagen type III				TGF-β1			
			+	++	+++	++++	+	++	+++	++++	-	+	++	+++
Normal	—	12	11	1	0	0	10	2	0	0	12	0	0	0
Model	—	11	0	1	1	9	0	1	2	8	0	1	1	9
BJRG	600	10	2	4	4	0	2	4	3	1	2	5	3	0
CPhGs	500	12	3	5	3	1	4	4	3	1	3	6	2	1
	250	9	1	4	3	1	2	4	2	1	1	5	2	1
	125	10	1	5	1	3	1	5	3	1	2	4	3	1
Total		64	18	20	12	14	19	20	13	12	20	21	11	12

### Collagen type I

**Fig. 4:**
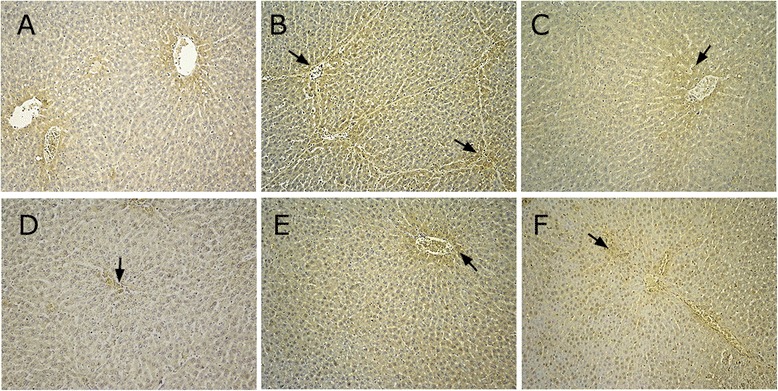
Expression of collagen type I in BSA-induced hepatic fibrosis in rats (immunohistochemical staining, magnification × 200). **a** normal group; **b** model group; **c** positive group; **d** CPhGs high dose group; **e** CPhGs middle dose group; **f** CPhGs low dose group

The normal group expressed collagen type I mainly in blood vessels and the portal area. The model group highly expressed collagen type I vascular fibrosis in portal areas. In the space of Disse, collagen type I staining was observed a streaks or as a patchy distribution. Collagen encased fibrous septa to form pseudolobule. In these experiments, fewer fibrous septa were formed for BJRJ (600 mg/kg) and different dose groups of CPhGs s (125, 250, and 500 mg/kg) [P_Col I_ = 0.002, P_Col I_ = 0.001, P_Col I_ = 0.023, and P_Col I_ = 0.044, respectively]. The semiquantitative results revealed that the expression of their collagen type I was significantly lower compared with the model group (Fig. 4).

### Collagen type III

**Fig. 5:**
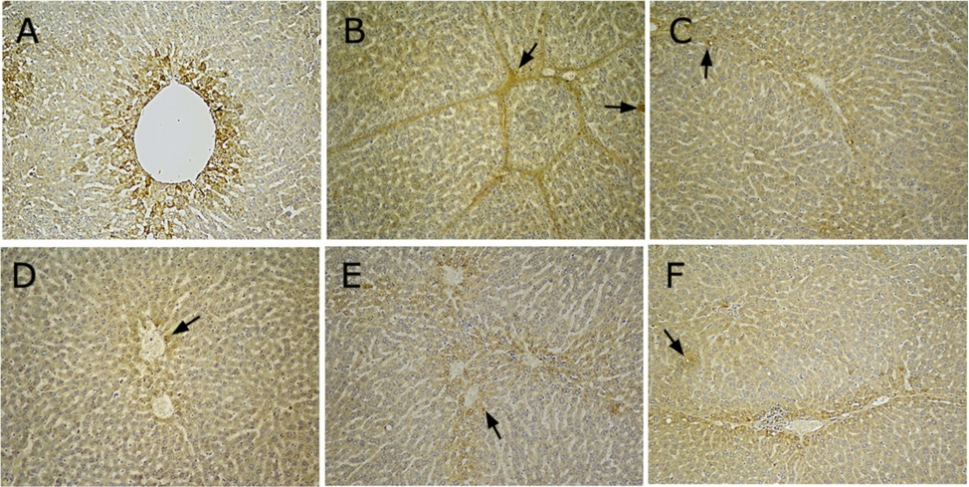
Expression of collagen type III in BSA-induced hepatic fibrosis in rats (immunohistochemical staining, magnification × 200). **a** normal group; **b** model group; **c** positive group; **d** CPhGs high dose group (500 mg/kg); **e** CPhGs middle dose group (250 mg/kg); **f** CPhGs low dose group (125 mg/kg)

Collagen type III was weakly expressed in the normal group, and peripheral areas surrounding the portal and hepatic veins displayed a small amount of fine yellow instead of continuous fibers (Fig. 5a). In the model group, collagen fibers exhibited wide and thick cords, indicating strong expression, mainly located in the portal and fibrous tissue areas (Fig. 5b).

Collagen fibers of BJRJ (600 mg/kg) and different dose groups of CPhGs (125, 250, and 500 mg/kg) were filamentous and distributed around the central vein and portal areas. Compared with the model group, the collagen fibers were significantly reduced, staining was pale and thin, and immunohistochemical staining was weakly positive (Fig. 5c–f). These semi quantitative results show that the positively expressed cells in the positive and different dose groups [*P*_Col III_ = 0.015, *P*_Col III_ = 0.001, *P*_Col III_ = 0.010, and *P*_Col III_ = 0.037, respectively] were different from those in the model group.

### TGF-*β*_1_

**Fig. 6:**
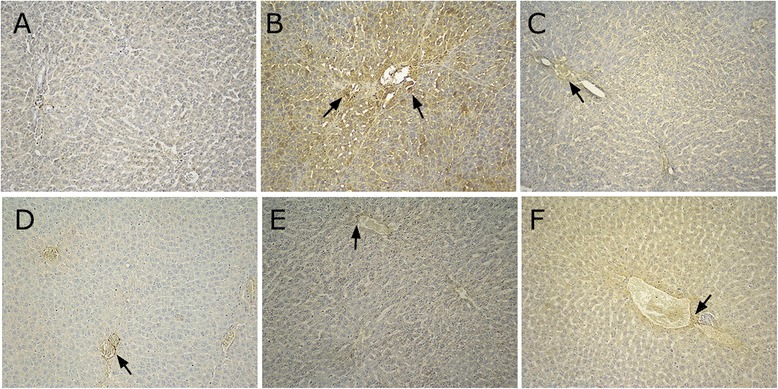
Expression of TGF-*β*_1_ in BSA-induced hepatic fibrosis in rats (immunohistochemical staining, magnification × 200). **a** normal group; **b** model group; **c** positive group; **d** CPhGs high dose group (500 mg/kg); **e** CPhGs middle dose group (250 mg/kg); **f** CPhGs low dose group (120 mg/kg)

There was little expression ofTGF-*β*_1_ in normal rat liver cells. Expression was limited to a small number of interstitial cells (Fig. 6a). In the model group, TGF-*β*_1_ expression was widely distributed in the portal area, fibrous spaces, hepatic stellate cells, inflammatory cells, the sinusoidal wall and cytoplasm. Aparticular portal area exhibited a strongly positive expression with brownish yellow staining (Fig. 6b).

There was a small amount of expression in the portal area and fibrous septa of BJRJ (600 mg/kg) and different dose groups of CPhGs (125, 250, and 500 mg/kg). The extent of positive staining in these groups was significantly reduced compared with that of the model group. Staining in the interstitial cells in the fibrous septa and the cytoplasm of inflammatory cells was decreased (Fig. 6c–f). Semi quantitative results revealed that BJRJ and different dose groups of CPhGs [*P*_TGF-*β*1_ = 0.001, *P*_TGF-*β*1_ < 0.001, *P*_TGF-*β*1_ = 0.009, *P*_TGF-*β*1_ = 0.004, respectively] were significant compared with the model group.

As mentioned earlier in the article, TGF-*β*_1_ is an important cytokine in the pathophysiology of liver fibrosis, stimulating the production of extracellular matrix [19]. We showed that the level of TGF-*β*_1_ increased in the model group in a manner consistent with the severity of liver fibrosis. The expression levels of TGF-*β*_1_ in liver were consistent with serum TGF-*β*_1_ levels. This indicates that CPhGs can significantly reduce liver fibrosis due to TGF-*β*_1_ expression by participating in the synthesis and degradation of ECM.

The expression of collagen type I, collagen type III, and TGF-*β*_1_ can detect the pathological process of hepatic fibrosis. Their expression levels in the treatment groups were significantly decreased, and illustrated that CPhGs can improve collagenase activity, maintaining the dynamic equilibrium of liver ECM synthesis and degradation, thus delaying and preventing the formation of liver fibrosis.

### NF-κB p65 and collagen I expression after drug intervention in HSC-T6 cells

**Fig. 7:**
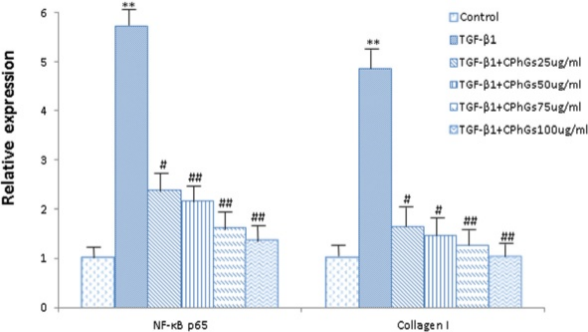
CPhGs down-regulated the expressions of NF-κB and collagen I mRNA in HSC-T6 cells (RT-PCR assay). Data were analyzed via one-way ANOVA followed by Bonferroni post-tests. Results are expressed as the mean ± SE. Notes the following: **P < 0.01 *vs.* control group; ^#^ *P* < 0.05 *vs.* TGF-β_1_ induced; ^##^ *P* < 0.01 *vs.* TGF-β_1_ induced

In order to characterize signals of hepatic fibrosis, two key regulatory genes in the liver were determined via RT-PCR assay. The data showed that tHSC-T6 cells from the control group had lower levels of NF-κB and collagen type I mRNA. Conversely, TGF-β_1_ induced HSC-T6 cells to markedly upregulate NF-κB (*P* < 0.01) and Col-I (*P* < 0.01) mRNA, with levels higher than those in the control group. In the presence of different concentrations of CPhGs , the results of NF-κB p65 [*P*_NF-κB_ = 0.001 for 100 ug/ml, *P*_NF-κB_ = 0.002 for 75 ug/ml, *P*_NF-κB_ = 0.007 for 50 ug/ml, and *P*_NF-κB_ = 0.012 for 25 ug/ml, respectively] and Col-I [*P*_Col-I_ = 0.006, *P*_Col-I_ = 0.009, *P*_Col-I_ = 0.014, *P*_Col-I_ = 0.019, respectively] showed reduced expressions of these mRNAs (Fig. 7).

### Western blot analysis of collagen I levels after drug intervention in HSC-T6 cells

**Fig. 8:**
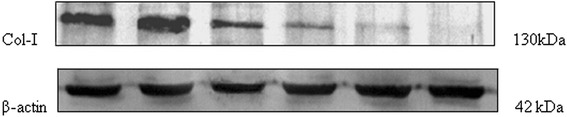
Collagen I protein expression. Protein samples were subjected to electro-transfer to a PVDF membrane, incubated with primary antibodies and anti-rabbit secondary antibodies conjugated with AP. Lane 1: normal; lane 2: TGF-β_1_ (5.0 ng/mL); lane 3: TGF-β_1_ + CPhGs (25ug/ml); lane 4: TGF-β_1_ + CPhGs (50 ug/ml); lane 5: TGF-β_1_ + CPhGs (75ug/ml); lane 6: TGF-β_1_ + CPhGs (100ug/ml)

Figure 8 shows the collagen I protein expression levels in HSC-T6 cells of the different experimental groups. The collagen I protein expression level was significantly decreased in the various dose groups of CPhGs (100 ug/ml, 75 ug/ml, 50 ug/ml, and 25 ug/ml) compared with the TGF-β_1_ group.

## Discussion

CPhGs is a phenylethanoid glycoside isolated and purified from rhizome of Cistanche, which is used as a traditional Chinese herbal medicine. In recent years, CPhGs had been shown to possess powerful ability to prevent liver injuries [20]. Therefore, we aimed to investigate whether CPhGs have inhibitory effects on hepatitis fibrosis by BSA induced hepatic fibrosis in rats. BJRG is commonly used as therapeutic drug for hepatic fibrosis in China. It is made from turtle shell. *Radix paeoniae rubra, Cordyceps sinensis, Radix isatidis,* and etc. have the effects of replenishing Qi and blood, relieving fatigue, softening nodes. In addition, previous studies show that it has the obvious function of blocking early liver fibrosis, inhibiting proliferation of fat storing cells, and reducing collagen synthesis [21]. Therefore, BJRG was used as a positive drug in this study.

The pathological changes of hepatic fibrosis in rats induced by BSA injections are similar to those in human portal cirrhosis [22]. CPhGs dose-dependently alleviated the degree of liver fibrosis and inhibited HSC transformation into myofibroblast-like cells, reduced the elevated levels of serum ALT, AST, HA, LN, CIV, TGF-*β*1 and the liver index, and markedly suppressed expression of collagen I, collagen III and TGF-*β*1 in liver tissue.

The stages of hepatic fibrosis are correlated with the serum levels of HA, LN and IV-C, which as markers may play a role in detecting the degree of hepatic fibrosis [23]. It has been reported that HA is the major resource of extracellular matrix. IV-C as the essential element of the basement membrane will be synthesized abundantly and deposit heavily in the earlier phases liver cirrhosis. The serum levels of LN and IV-C are the indexes of the turnover rate of the basement membrane and show the degree of fibrosis in the portal area and sinusoidal capillaries [24]. PC III is a marker in the diagnosis of hepatic fibrosis and early cirrhosis, but its sensitivity and specificity are not high, and there is no significant difference between the various stages of fibrosis in many references [24, 25]. This study obtained a similar result.

In addition, the H&E and Masson’s trichrome stained section observations exhibit normal liver tissues with distinct hepatic lobules and hepatic sinusoids. The liver tissue structure in the model group was disordered, and the liver tissue and hepatic sinusoid were replaced by a large amount of connective tissue. However, significantly improvement was observed in the treatment groups compared with the model group.

Importantly, collagen type I and collagen type III expression play essential roles in the development of hepatic fibrosis, the blocking of which can prevent and treat hepatic fibrosis. Therefore, the generation and deposition in the liver tissue of collagen type I and collagen type III could serve as an important determinant of anti-hepatic fibrosis efficacy. TGF-*β*_1_ is also an important profibrogenic cytokine in liver injury and it is biologically active with multiple pharmacological actions [26]. A balance among these actions is required to maintain tissue homeostasis. The aberrant expression of TGF-*β*_1_ is involved in the pathogenesis of liver diseases [27, 28]. It is known that TGF-*β*_1_ is a crucial cytokine that is involved in the early stages of liver fibrosis. Oxidative stress triggers TGF-*β*_1_, resulting in the latter stimulating ECM production and deposition [29]. Therefore, one of the effective strategies to produce anti-hepatic fibrosis drug is to identify anti-TGF-*β*_1_ agents. Immunohistochemical analysis showed that the expressions of collagen type I, collagen type III and TGF-*β*_1_ could detect the pathological process of hepatic fibrosis. The expressions of the collagen type I, collagen type III and TGF-*β*_1_ in the treatment groups are decreased, which were significantly lower in the high dose CPhGs treatment group in particular, and suggested that CPhGs is an effective collagen type I, collagen type III and TGF-*β*1 inhibitor. Presumably, CPhGs can improve collagenase activity, maintaining the dynamic equilibrium of ECM synthesis and degradation, thus delaying and preventing the formation of liver fibrosis.

CPhGs not only could ameliorate BSA-induced hepatic fibrosis in rats, but also might be associated with inhibiting the activation of HSC in vitro*.* HSC activation is thought to represent the crucial step of fibrogenesis. In this study, the results illustrated that administration of CPhGs from 25 to 100 ug/ml remarkably attenuated the decreased NF-κB p65, collagen I mRNA expression, and collagen I protein expression in HSC.

NF-κB plays an important role in modulating the immune response to infection or stimuli [30]. Buildup of NF-κB in liver cells can result in the recruitment of inflammatory cytokines/mediators, thus inducing fibrosis development [31, 32]. Moreover, collagen is also a sensitive index that reflects the fibrosis level and accounts for about 50 % of the total protein in fibrous liver [33]. As a result, we postulated that the molecular mechanism against hepatofibrosis is linked to CPhGs-mediated inactivation of NF-κB expression, in which the benefit contributes to synergistic roles of attenuating immunotoxicity and inflammation stress in BSA-lesioned liver tissue, further correcting dysmetabolism to ameliorate liver functions.

## Conclusions

In conclusion, our studies indicate that CPhGs significantly attenuate the extent of hepatic fibrosis induced by BSA in rats. Its mechanism may at least partially be due to the inhibitory effect of CPhGs on the composition of ECM and stimulation of the degradation of ECM, and/or by directly inhibition of the synthesis of collagen type I, collagen type III and the expression of TGF-*β*_1_. Therefore, we expected that CPhGs can be used in health care products or in clinical medications for prevention of human liver fibrosis. Future studies are required to establish the efficacy of CPhGs as a potent anti-hepatic fibrosis drug.

## Abbreviations

CPhGs: Phenylethanol glycosides from cistanche
BSA: Bovine serum albumin
HSC-T6: Hepatic stellate cells
Hyp: Hydroxyproline
BJRG: Compound Biejiarangan tablets
TGF-*β*_1_: Transforming growth factor *β*_1_
ALT: Alanine aminotransferase
AST: Aspartate aminotransferase
HA: Hyaluronic acid
LN: Laminin
PC III: Type III precollagen
IV-C: Type IV collagen
NF-κB: Nuclear factor kappa-light-chain-enhancer of activated B cells
RT-PCR: Reverse transcriptase polymerase chain reaction
SDS-PAGE: Sodium dodecyl sulfate polyacrylamide gel electrophoresis
